# Bilateral Spontaneous Resolution of Traumatic Epidural Hematoma: A Case Report and Literature Review

**DOI:** 10.7759/cureus.39379

**Published:** 2023-05-23

**Authors:** Abdulsalam Aleid, Basmah S Alzahrani, Murtadha H Alameer, Abdulsalam J Alhassan, Ibrahim Alahmed

**Affiliations:** 1 Neurosurgery Department, King Faisal University, Al-Hofuf, SAU; 2 Neurosurgery Department, King Fahad Hospital, Al-Hofuf, SAU; 3 Health Rehabilitation, Armed Forces Center for Health Rehabilitation, Taif, SAU

**Keywords:** accidental head trauma, literature review of disease, rare case report, spontaneous resolution, epidural hematoma

## Abstract

Epidural hematomas (EDHs) are a neurosurgical emergency characterized by the accumulation of blood in the epidural space surrounding the dura mater. Spontaneous resolution of EDH is an exceptionally rare occurrence, with only 16 cases reported in the medical literature where resolution occurred within 24 hours of onset. In this case report, we present a unique instance of a chronic EDH that spontaneously resolved over a period of seven months. This case adds to the scientific literature by highlighting an extremely prolonged duration of spontaneous EDH resolution, which, to our knowledge, has not been previously documented.

A 59-year-old male suffered a head injury following a fall. He presented with a progressively worsening headache and nausea, raising concerns for a potential EDH. A computed tomography (CT) scan confirmed the presence of a large right parietal EDH measuring 58 × 23 × ​​​​​​​17 mm and a large left frontoparietal EDH measuring 90 × 20 ×​​​​​​​ 12 mm. These findings were crucial in establishing the primary diagnosis and guiding subsequent interventions. Upon diagnosis of the EDHs, the patient received conservative treatment and was closely monitored. Over a period of seven months, follow-up imaging revealed complete resolution of both EDHs, with restoration of normal midline structures and ventricular sizes. Notably, this represents the longest duration of spontaneous EDH resolution reported in the literature. We attribute this uncommon outcome to the activation of endogenous fibrinolytic pathways, which are responsible for dissolving blood clots and hematomas. In addition, the formation of new collateral blood vessels around the hematoma may help facilitate its resolution.

This case underscores the significance of early recognition and vigilant monitoring of EDH cases. While immediate surgical intervention remains essential in most instances, conservative management can be considered in select cases. Our report demonstrates the possibility of spontaneous resolution of EDHs over an extended period, emphasizing the importance of continued observation and appropriate management. By shedding light on this rare occurrence, this case report contributes to the limited existing literature on the topic, providing valuable insights and adding to our understanding of EDH management.

## Introduction

Epidural hematomas (EDHs) are blood accumulations in the epidural space separating the dura mater from the skull that could be the result of a traumatic event. They constitute a neurosurgical emergency that neurosurgeons worldwide never ignore. EDH is among the most prevalent medical emergencies treated by neurosurgeons. The standard of care for EDH causing significant brain compression and mass effects is urgent surgical evacuation [1]. However, isolated asymptomatic EDH devoid of mass effect may be closely monitored with serial neurological examinations and imaging for the possibility of spontaneous resolution or expansion. Typically, EDH resolves within two weeks [1], and resolution within 24 hours is extremely uncommon, with only a handful of cases reported [2,3]. We present a case of spontaneous resolution of chronic EDH over seven months, the longest duration reported in the medical literature. We attribute this uncommon outcome to potential communication between the epidural and epicranial spaces, allowing the hematoma to gradually self-evacuate. This case adds to the scant literature on spontaneous EDH resolution and emphasizes the significance of early diagnosis and treatment. In addition, it emphasizes that initial conservative management and close monitoring may be advantageous in particular cases. Before deciding between immediate evacuation and watchful waiting, neurosurgeons should consider EDH's neurological status, volume, and mass effect. Despite its rarity, the possibility of delayed spontaneous resolution must also be considered. In conclusion, we report a rare case of spontaneous resolution of chronic EDH over seven months. This increases our knowledge of EDH pathology and treatment. Although some cases can be initially managed and monitored conservatively, prompt diagnosis and treatment are still essential. We hope to raise awareness of this peculiar outcome by disseminating this report.

## Case presentation

**Figure 1:**
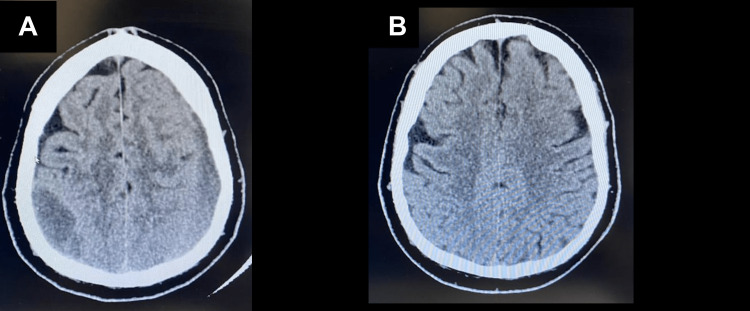
Axial CT scan of the brain: (A) bilateral parietal hypodense fluid collection and (B) bilateral epidural hematoma resolution. CT: computed tomography

**Figure 2:**
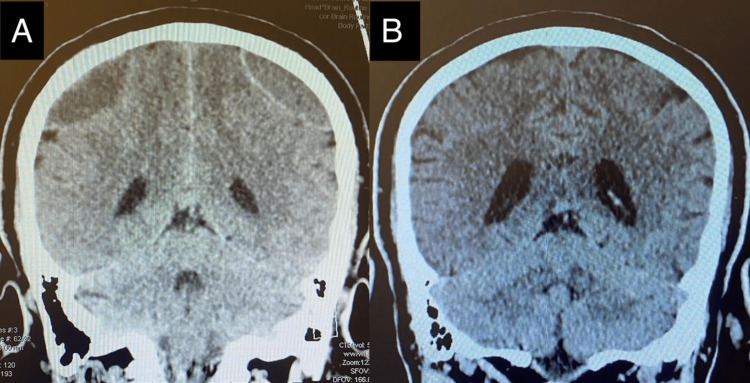
Coronal CT scan of the brain: (A) bilateral parietal hypodense fluid collection and (B) the resolution of bilateral EDH. CT: computed tomography, EDH: epidural hematoma

After a trivial fall, a 59-year-old male patient presented to our care approximately two weeks later, complaining of recurrent headaches and dizziness that had been progressively worsening since the incident. The patient reported experiencing pain and headache on both sides of the head, primarily above both ears, with a gradual escalation of symptoms. Notably, there were no observed neurological deficits during the neurological assessment and examination. The scalp exhibited mild tenderness since the fall. The patient had no significant history of previous trauma, making this event the first of its kind. A thorough neurological examination conducted by a neurosurgeon revealed no abnormalities. The initial computed tomography (CT) scan, performed two weeks after the fall, unveiled a large right parietal epidural hematoma (EDH) measuring 58 × 23 × 17 mm and a large left frontoparietal EDH measuring 90 × 20 × 12 mm (Figure 1A, Figure 2A). Notably, no skull fractures were observed. Based on these CT scan findings, the diagnosis was revised to chronic EDH. The patient exhibited a Glasgow Coma Scale (GCS) score of 15/15 during the neurological assessment, with no evidence of anisocoria. The patient had no chronic illnesses, was not taking any medications, and was not on any anticoagulants. Despite being in stable condition without complicating factors, a seemingly minor fall resulted in the development of bilateral epidural hematomas (EDHs). Following the accident, there were no reports of loss of consciousness or seizures. Cranial nerve function and motor/sensory systems were intact upon examination. Given the stable condition of the patient and the absence of complicating factors, a conservative approach was chosen, and the patient was closely monitored. Seven months later, a repeat CT scan was performed due to the patient's noncompliance with follow-ups, revealing a complete resolution of the hematoma (Figure 1B, Figure 2B). These clinical findings, including the absence of neurological deficits, guided the diagnosis and subsequent management decisions. The patient's medical history, family history, and psychosocial information were unremarkable, and there was no relevant genetic information. The initial approach involved conservative management and close monitoring, which eventually led to the spontaneous resolution of the EDHs over a seven-month period. To ensure patient privacy, the patient's personal details have been omitted from this report.

Diagnostic methods

The patient underwent a thorough physical examination to assess the neurological status and identify any abnormalities. No neurological deficits were observed during the examination. In addition, laboratory tests including coagulation parameters were performed to evaluate the patient's overall health and assess any underlying conditions that could contribute to the epidural hematoma. All laboratory results were within normal limits.

Imaging

A CT scan of the brain was conducted approximately two weeks post-fall, revealing the presence of a large right parietal epidural hematoma measuring 58 × 23 × 17 mm and a large left frontoparietal epidural hematoma measuring 90 × 20 × 12 mm. These imaging findings supported the diagnosis of chronic epidural hematoma. The main diagnostic challenge in this case was the patient's inconsistent compliance with follow-up appointments, which limited the ability to precisely determine the timeline of resolution and hindered the comprehensive assessment of the hematoma. The primary diagnosis in this case is chronic epidural hematoma (EDH). The patient presented with recurrent headaches, dizziness, and CT scan findings consistent with the presence of bilateral EDHs. Other potential diagnoses that were considered include acute epidural hematoma, subdural hematoma, and intracerebral hemorrhage. However, these alternative diagnoses were ruled out based on the clinical presentation, imaging findings, and absence of additional neurological symptoms. Prognostic characteristics specific to the resolution of the chronic epidural hematoma in this case were indeterminate due to the patient's unreliable nature and lack of regular follow-up appointments. However, it is noteworthy that the hematoma resolved over a period of seven months without surgical intervention. The patient no longer reported headaches or dizziness, and physical examinations, laboratory tests, and subsequent imaging indicated normal findings, suggesting a favorable prognosis. It is important to note that this diagnostic assessment provides an overview of the methods used to diagnose chronic epidural hematoma, the challenges encountered, the specific diagnosis, and considerations of other potential diagnoses. Additionally, it highlights the prognostic characteristics associated with the resolution of the hematoma in this particular case.

Therapeutic intervention

In this case, pharmacologic intervention refers to the conservative management approach adopted for the treatment of chronic epidural hematoma. The patient received nonsurgical treatment, which involved close monitoring and symptomatic management of associated symptoms. Surgical intervention was not performed in this case due to the stable condition of the patient, the absence of neurological deficits, and the successful resolution of the hematoma through conservative management. The specific dosages, strengths, and durations of pharmacologic interventions were not mentioned in the provided information. However, conservative management typically involves close monitoring of the patient's condition, including regular neurological assessments and imaging studies. Symptomatic management may involve the administration of analgesics or other medications to address pain and associated symptoms. No changes in therapeutic interventions were mentioned in the given information. However, it is important to note that the successful resolution of the chronic epidural hematoma over a period of seven months without the need for surgical intervention indicates the efficacy of the conservative management approach. The patient's compliance with follow-up appointments and adherence to the prescribed treatment plan would have been crucial for the therapeutic interventions to remain consistent and effective.

The resolution duration of the epidural hematoma in our case remains uncertain due to the patient's inconsistent compliance with follow-up appointments at our clinic. Despite this challenge, it is important to acknowledge that the patient initially presented with recurring headaches and dizziness, prompting a brain CT scan that incidentally detected a chronic EDH. Remarkably, after a period of seven months, the hematoma resolved without any surgical intervention. It is worth noting that the patient's unreliability in attending regular follow-ups hindered the ability to precisely determine the exact timeline of resolution. Nevertheless, the patient reported no ongoing complaints of headaches or dizziness, and all vital signs and physical examinations were within normal limits. Additionally, laboratory tests, including coagulation parameters, were unremarkable. During the follow-up period, the patient received two doses of the COVID-19 vaccine. Notably, the initial hospitalization lasted for two days, during which no surgical procedures were performed.

The timeline of patient events and findings is summarized in Table 1.

**Table 1:** Timeline of patient events and findings. The table provides a chronological overview of the episode of care for the patient with a chronic EDH. It outlines the key events and findings during various stages of the patient's journey. EDH: epidural hematoma, CT: computed tomography, GCS: Glasgow Coma Scale, COVID-19: coronavirus disease 2019

Timeline	Events and findings
Pre-fall	No significant medical history, chronic illnesses, medications, or anticoagulant use reported by the patient
	No history of previous trauma prior to the fall
	Patient in good health and stable condition
Day of fall	Trivial fall occurred
Approximately two weeks post-fall	Patient presents with recurrent headaches and dizziness, progressively worsening
	Scalp tenderness observed since the fall
	Neurological examination shows no abnormalities
	Initial CT scan reveals a large right parietal EDH measuring 58 × 23 × 17 mm
	Initial CT scan reveals a large left frontoparietal EDH measuring 90 × 20 × 12 mm
	No skull fractures observed
	Diagnosis revised to chronic EDH based on CT scan findings
	GCS score of 15/15 and no evidence of anisocoria
	Patient in stable condition with intact cranial nerves and motor/sensory systems
	Conservative approach chosen for management, patient closely monitored
Seven months post-fall	Repeat CT scan performed due to patient noncompliance with follow-ups
	Repeat CT scan reveals complete resolution of hematoma
	Patient no longer complains of headaches or dizziness
	Vital signs and physical examination are normal
	Laboratory tests, including coagulation parameters, are within normal limits
	Patient receives two doses of the COVID-19 vaccine
	Initial hospitalization lasted for two days, no surgical procedures performed

Table 1 provides a chronological overview of the episode of care for the patient with a chronic epidural hematoma (EDH). It outlines the key events and findings during various stages of the patient's journey, including pre-fall, the day of the fall, and approximately two weeks and seven months post-fall. The timeline highlights important details such as the patient's presentation of recurrent headaches and dizziness, neurological examinations, initial CT scan findings revealing the size of the EDHs, the absence of skull fractures, the diagnosis of chronic EDH, GCS score, intact cranial nerves, conservative management approach, patient noncompliance with follow-ups, and subsequent resolution of the hematoma. Additionally, it includes relevant information about the patient's vital signs, physical examination, laboratory tests, COVID-19 vaccination, and the duration of the initial hospitalization. This comprehensive timeline serves to provide a clear understanding of the patient's clinical course and the resolution of the chronic EDH over time.

## Discussion

Although many theories have been put forth, the spontaneous resolution of EDH has not yet been fully explained. However, the literature has reported 16 cases with resolutions in fewer than 24 hours as shown in Table 2.

**Table 2:** Cases of spontaneous resolution of epidural hematoma. Note: The size of hemorrhage is given in mm. Time to resolution is given in h. mm: millimeters, h: hours, ICP: intracranial pressure, M: male, F: female, NA: not applicable/available, m: month(s)

Author, year	Age/sex	Location of hemorrhage	Size of hemorrhage	Skull fracture	Increased ICP	Epicranial hematoma	Time to resolution
Aoki et al., 1988 [2]	8/M	Left temporal	15 mm	Yes	None	Increase	23 h
Aoki et al., 1988 [2]	17/M	Left occipital	10 mm	Yes	None	Increase	5 h
Servadei et al., 1989 [3]	65/M	Right parietal	15 mm	Yes	Yes	NA	4 h
Eom et al., 1993 [4]	17/M	Right occipital, posterior fossa	20 mm	Yes	None	NA	12 h
Malek et al., 1997 [5]	17 m/M	Right temporal	8 mm	Yes	None	Increase	20 h
Ugarriza et al., 1999 [6]	3.5/M	Left temporal	15 mm	Yes	None	Increase	16 h
Ugarriza et al., 1999 [6]	43/M	Right temporal	22 mm	Yes	None	Increase	6 h
Celikoğlu et al., 2002 [7]	48/M	NA	NA	NA	NA	NA	1 h
Celikoğlu et al., 2002 [7]	8/M	Right temporal	10 mm	Yes	None	Increase	1 h
Celikoğlu et al., 2005 [7]	34/M	Bilateral posterior fossa	NA	Yes	Yes	Increase	21 h
Kang et al., 2005 [8]	2.5/F	Left parietal	10 mm	Yes	None	Increase	10 h
Ugarriza et al., 2009 [6]	13/F	Right temporal	18 mm	Yes	None	Increase	16 h
Celikoğlu et al., 2011 [7]	27/M	Right temporal	12 mm	Yes	None	NA	3 h
Gulsen et al., 2013 [9]	4/F	Right temporal	17 mm	No	None	NA	12 h
Aydemir et al., 2016 [10]	11 m/M	Right parietal	9 mm	Yes	NA	Yes	3 h
Manne et al., 2019 [11]	M/20	Right parietal, anterior temporal convexity	NA	Yes	NA	Increase	7 h
Present case	59/M	Right parietal, anterior temporal convexity	Right parietal area (58 × 23 × 17 mm), left frontoparietal area (90 × 20 × 12 mm)	No	None	NA	7 m

Epidural hematomas and other intracranial hemorrhages can have a range of resolution times and natural courses. It is essential to comprehend these traits for the treatment and prognosis of patients. The normal progression of epidural hematomas, their resolution period, and their hyper-/hypo-/iso-intense phases are discussed here. An epidural hematoma is a collection of blood between the dura mater, the meninges' outer layer, and the skull's interior surface. It frequently happens as a result of a traumatic head injury, which causes an artery to rupture, most frequently, the middle meningeal artery. Initial bleeding may result in the hematoma expanding quickly, raising the intracranial pressure and raising the risk of potentially fatal complications. There are several phases to an epidural hematoma's natural course. Blood quickly builds up in the epidural space in the acute bleeding phase that follows the trauma. This stage is marked by the hematoma's quickening growth, which raises intracranial pressure. The symptoms can be severe and advance quickly during this stage. After the acute stage, there is a time of stabilization. The clotting process starts during this stage, and the hematoma may stop growing. The stabilization period can last for a short while or for several days. The length of this phase depends on the size and location of the hematoma as well as the particular patient's circumstances. In some instances, a spontaneous resolution phase may follow the stabilization phase. The hematoma may gradually resorb and degrade, which would cause it to get smaller over time. The body's natural healing processes, which include the dissolution of the blood clot and reabsorption of the blood's constituent parts, are responsible for this resolution.

An epidural hematoma's time to resolve can also differ significantly. Complete resolution may take weeks or months in some cases, while residual blood may last longer in others. The size and location of the hematoma, the patient's age and general health, and the existence of any underlying coagulopathies or vascular anomalies are all factors that can affect resolution. Monitoring the development of an epidural hematoma requires the use of imaging techniques such as computed tomography (CT) or magnetic resonance imaging (MRI). These scans can identify changes in the hematoma's size, density, and intensity, which can reveal important details about its natural progression and resolution. Imaging studies may show periods of hyperintensity, hypointensity, or isointensity as an epidural hematoma progresses. These alterations may represent the hematoma's developmental stages. Areas of increased signal intensity, or hyperintensity, may represent recent bleeding or persistent expansion. Hypointensity can indicate the resolution or degradation of the hematoma by indicating a decrease in signal intensity. Areas with signal intensity comparable to surrounding tissues are referred to as isointensity, which denotes stability or a static state of the hematoma. It is significant to remember that not all epidural hematomas resolve on their own. In some circumstances, surgery is necessary, especially when the hematoma is large, has a significant mass effect, or is linked to neurological decline. The patient's clinical condition, the results of any imaging tests, and the level of available neurosurgical expertise all play a role in the decision to pursue conservative management or surgical intervention. The resolution of our case might occur in less time than seven months. The precise moment is unknown. Once the patient was relieved that no intervention could be made, they continued with clinic care after their symptoms subsided. We draw the conclusion that the resolution took place over a period of seven months based on that and the information provided, which has never been reported in cases similar to ours [2-11]. Ten of the patients under 18 were children, and six were adults. All but two of the cases involved skull fractures, including our case.

According to Aoki et al. [2], a skull fracture improves prognosis because it connects the epicranial and epidural spaces [3]. Rapid spontaneous resolution without increased intracranial pressure was reported in two EDH cases with fractures that included the external auditory canal [2,4]. Gülşen et al. [9] reported a case of spontaneous resolution without skull fracture in a four-year-old child. It was assumed that the hematoma, in this case, was connected to the sutures' opening because it was close to the cranial sutures [4], postulating that after head trauma, the increased pressure causes blood and serous fluid in the epicranial space to flow through the fracture into the epidural space, where they reverse direction and flow back into the epicranial space through the fracture [5]. However, this process takes around 18 hours to complete, which is not long enough to account for the seven-month resolution in our case. Furthermore, increased intracranial pressure was thought to cause the counterflow of EDH into the epicranial area [2,6]. However, only two cases documented in the literature have elevated intracranial pressure. Another theory holds that bleeding from the diploic space spreads into the epidural and subgaleal spaces through a fracture in the early stages of trauma and that passage into the subgaleal space from the epidural space occurs as a result of the pulsatile effect of the brain [2]. In the case of spontaneous resolutions without ICP rising, the hemorrhage into the subgaleal space may have been decompressed [3,7], leading to a relatively lower pressure in the subgaleal space compared to the intracranial pressure. In 10 cases previously documented in the literature, an increase in hematoma in the epicranial region was reported (Table 2). Although surgical evacuation is a settled opinion in managing EDHs, the opinion of conservative follow-up has made its way into the literature. The primary causes of this can be summed up as follows: more intensive care units than before, allowing for easier close follow-up of patients with head injuries, 24-hour availability of neurosurgeon staff in many health centers, and convenient access to CT scans, which is crucial for the diagnosis and care of these patients.

After the initial report in 1981, more cases of acute EDHs have resolved quickly on their own, which may support the recommendation for conservative follow-up [5,7]. The pressure-induced redistribution caused by brain swelling is one of the likely mechanisms for spontaneous resolution; however, the dissipation of hematomas appears more difficult because of tenacious adhesions between the dura mater and the skull [8-10]. Another theory suggested that the extracranial blood leak could be caused by a pressure gradient that results in increased epicranial subgaleal interstitial pressure following injury [12]. Blood leaks back when the interstitial subgaleal pressure decreases [13]. About 18 hours is needed to complete this. Due to this, this theory appears to fall short of fully describing the underlying mechanism in all cases, especially those that resolved in under 18 hours. We lack precise information on the potential impact of the volume of the skull's contents on resolution [2-11]. We believe filling the skull with its contents (brain, cerebrospinal fluid, etc.) may be important in this resolution process [12,13]. Because a hematoma would have more epidural space than the brain could hold, brain atrophy could prevent spontaneous resolution from occurring quickly. Only one case of advanced age (65 years old) is documented in the literature [8]. The longest resolution time for pediatric cases was 72 hours [6], and the shortest was just one hour [10].

In our case, the thickness was 17 mm, but the GCS was 15 and the total blood volume was less than 30 cm^3^. The resolution would have possibly been visible sooner if the CT scan had been performed sooner. In this case, a patient of unknown age and gender was taken to the hospital after being involved in a motorcycle accident and complaining of headache and lightheadedness. The scalp was slightly tender, but no other abnormalities or neurological deficits were found during the physical examination. No abrasions or erythema were visible in the trauma area; only edema was visible, and the patient had no known medical conditions or histories. The patient did not have any skull fractures. Even without surgical intervention, the hematoma completely resolved after seven months, according to a subsequent CT scan. During the patient's hospital stay, there were no notable or unusual laboratory findings, and neither the patient's GCS score nor anisocoria was noted during an examination.

A connection between the epidural and epicranial spaces created by a fracture or cranial sutures might play a significant role in the rapid spontaneous resolution of an EDH, according to the case being discussed and other cases that have been previously published. There are several potential explanations for the spontaneous resolution of an EDH in our case, including the activation of endogenous fibrinolytic pathways, which are responsible for dissolving blood clots and hematomas. In addition, the formation of new collateral blood vessels around the hematoma may help facilitate its resolution. It is also possible that the EDH was initially smaller than it appeared on the initial CT scan and subsequently underwent self-resolution over time. However, further research is needed to fully understand the underlying mechanisms of spontaneous EDH resolution.

Our case report highlights the rare occurrence of a traumatic EDH. Although the mechanism of injury did not seem severe and the patient had no comorbid conditions, the injury itself proved to be life-threatening, requiring emergent neurosurgical evacuation of the hematomas. However, due to the patient's noncompliance with further management and follow-ups, the EDH has spontaneously resolved without surgical intervention. This case highlights the need for close monitoring of patients with EDH and the possibility of a favorable outcome without surgical intervention. We attribute this uncommon outcome to potential communication between the epidural and epicranial spaces, allowing the hematoma to gradually self-evacuate. This case furthers our understanding of the pathophysiology and treatment of EDH. Although evacuation is essential for hematomas causing mass effects, asymptomatic or small chronic hematomas may be initially managed and monitored conservatively. However, complications and surgical risks must be weighed.

We hope that this report generates additional discussion on EDH management strategies. In addition, this comparison is intended to highlight the contrasting timeline of resolution between our case and previously reported cases. By showcasing this difference, we sought to underscore the exceptional nature of our patient's condition and the need for further investigation into the factors contributing to prolonged spontaneous resolution. In previously reported cases, spontaneous resolution of epidural hematomas occurred within a few days to a few weeks. In our case, however, the hematoma resolved over a period of several months. This suggests that there may be factors that contribute to prolonged spontaneous resolution that are not yet fully understood. Further investigation into these factors is warranted. This could lead to the development of new treatments that could help patients with epidural hematomas who are not candidates for surgery or who do not respond to surgical treatment. This case report adheres to the CARE Checklist of 2013 and does not need ethical approval or informed consent from the patient as no personal data is mentioned and the patient's identity is anonymous. It provides a concise summary of the patient's medical history, presenting symptoms, and diagnostic findings. The findings contribute to our limited understanding of the underlying mechanism behind the spontaneous resolution of EDH. By comparing our case to previously reported cases, we shed light on the unique nature of the spontaneous resolution observed in our patient, highlighting the need for further research and exploration in this area. The report emphasizes the rarity of such cases and their potential implications for the management and treatment of EDH. The report acknowledges the importance of early recognition, monitoring, and appropriate management of cases and emphasizes the need for further evaluation.

## Conclusions

This case report describes a spontaneous bilateral resolution of an EDH over seven months, which has not previously been reported in similar cases resulting from patient noncompliance. The medical literature documents 16 cases with resolution in less than 24 hours, of which 10 were pediatric, six were adult, and all but two were associated with skull fractures, including our case. Numerous hypotheses have been put forward to explain why EDH spontaneously resolves. Conservative follow-up has gained ground in the literature due to the availability of intensive care units, neurosurgeon staff, and easy access to CT scans. This case report is significant due to the rarity of spontaneous bilateral resolution of EDH over seven months and its contribution to our limited understanding of the underlying mechanism of EDH spontaneous resolution. This comparison highlights the difference in spontaneous resolution of epidural hematomas between our case and previously reported cases, suggesting that there may be factors contributing to prolonged spontaneous resolution. Further investigation is warranted to develop new treatments. The case report emphasizes the significance of conservative follow-up in EDH management and the need for additional research to better comprehend the mechanism of spontaneous resolution.
